# Silver(I) and Copper(II) Complexes of 1,10-Phenanthroline-5,6-Dione Against *Phialophora verrucosa*: A Focus on the Interaction With Human Macrophages and *Galleria mellonella* Larvae

**DOI:** 10.3389/fmicb.2021.641258

**Published:** 2021-04-27

**Authors:** Marcela Q. Granato, Thaís P. Mello, Renata S. Nascimento, Marcos D. Pereira, Thabatta L. S. A. Rosa, Maria C. V. Pessolani, Malachy McCann, Michael Devereux, Marta H. Branquinha, André L. S. Santos, Lucimar F. Kneipp

**Affiliations:** ^1^Laboratório de Taxonomia, Bioquímica e Bioprospecção de Fungos (LTBBF), Instituto Oswaldo Cruz (IOC), Fundação Oswaldo Cruz (FIOCRUZ), Rio de Janeiro, Brazil; ^2^Laboratório de Estudos Avançados de Microrganismos Emergentes e Resistentes (LEAMER), Instituto de Microbiologia Paulo de Góes, Universidade Federal do Rio de Janeiro (UFRJ), Rio de Janeiro, Brazil; ^3^Laboratório de Citotoxicidade e Genotoxicidade (LaCiGen), Instituto de Química, UFRJ, Rio de Janeiro, Brazil; ^4^Laboratório de Microbiologia Celular, IOC/FIOCRUZ, Rio de Janeiro, Brazil; ^5^Department of Chemistry, Maynooth University, National University of Ireland, Maynooth, Ireland; ^6^Center for Biomimetic and Therapeutic Research, Focas Research Institute, Technological University Dublin, Dublin, Ireland

**Keywords:** 1,10-phenanthroline-5,6-dione, chromoblastomycosis, dematiaceous fungi, antifungal activity, cellular interaction

## Abstract

*Phialophora verrucosa* is a dematiaceous fungus that causes mainly chromoblastomycosis, but also disseminated infections such as phaeohyphomycosis and mycetoma. These diseases are extremely hard to treat and often refractory to current antifungal therapies. In this work, we have evaluated the effect of 1,10-phenanthroline-5,6-dione (phendione) and its metal-based complexes, [Ag (phendione)_2_]ClO_4_ and [Cu(phendione)_3_](ClO_4_)_2_.4H_2_O, against *P. verrucosa*, focusing on (i) conidial viability when combined with amphotericin B (AmB); (ii) biofilm formation and disarticulation events; (iii) *in vitro* interaction with human macrophages; and (iv) *in vivo* infection of *Galleria mellonella* larvae. The combination of AmB with each of the test compounds promoted the additive inhibition of *P. verrucosa* growth, as judged by the checkerboard assay. During the biofilm formation process over polystyrene surface, sub-minimum inhibitory concentrations (MIC) of phendione and its silver(I) and copper(II) complexes were able to reduce biomass and extracellular matrix production. Moreover, a mature biofilm treated with high concentrations of the test compounds diminished biofilm viability in a concentration-dependent manner. Pre-treatment of conidial cells with the test compounds did not alter the percentage of infected THP-1 macrophages; however, [Ag(phendione)_2_]ClO_4_ caused a significant reduction in the number of intracellular fungal cells compared to the untreated system. In addition, the killing process was significantly enhanced by post-treatment of infected macrophages with the test compounds. *P. verrucosa* induced a typically cell density-dependent effect on *G. mellonella* larvae death after 7 days of infection. Interestingly, exposure to the silver(I) complex protected the larvae from *P. verrucosa* infection. Collectively, the results corroborate the promising therapeutic potential of phendione-based drugs against fungal infections, including those caused by *P. verrucosa*.

## Introduction

*Phialophora verrucosa* is a well-known chromoblastomycosis etiological agent that may also cause other cutaneous and subcutaneous diseases as well as disseminated infections, such as phaeohyphomycosis and mycetoma (Turiansky et al., 1995; Tong et al., 2013; Queiroz-Telles et al., 2017). *P. verrucosa* has been observed in patients suffering with distinct illnesses, including keratitis, endophthalmitis, sinusitis, osteomyelitis, and endocarditis (Lundstrom et al., 1997; Hofmann et al., 2005; Campos-Herrero et al., 2012). Infections caused by this melanized filamentous fungus can affect both immunocompetent and immunosuppressed individuals (Lundstrom et al., 1997; Qiu et al., 2019). To date, there is no gold standard therapy for these incapacitating and neglected diseases. Overall, the infections caused by *P. verrucosa* tend to chronicity and hinder treatments, leading to recurrence and resistance to available conventional therapies (Li et al., 2014; Brito and Bittencourt, 2018). This scenario has emphasized the necessity for the development of new drugs against *P. verrucosa* infections, to be administered either alone or in combination with established antifungal agents.

Metal-based drugs have attracted enormous attention due to their pharmacological effects, which includes anticancer, anti-inflammatory, antioxidant, and antimicrobial activities (Mjos and Orvig, 2014; Anthony et al., 2020). In fact, inorganic medicinal chemistry has advanced in recent years, providing an alternative therapeutic approach to the purely organic antimicrobial agents (Lemire et al., 2013; Viganor et al., 2017; Claudel et al., 2020). Several studies have shown that 1,10-phenanthroline (phen) and its derivatives, as ligands coordinated to transition metals, exhibit potent antifungal activity against *Candida albicans* and non-*albicans Candida* species, such as *C. tropicalis*, *C. krusei*, *C. glabrata*, and *C. haemulonii* (Geraghty et al., 2000; McCann et al., 2000, 2012; Coyle et al., 2003; Hoffman et al., 2015; Gandra et al., 2017, 2020). In a previous study, we showed that 1,10-phenanthroline-5,6-dione (phendione) and its silver(I) and copper(II) complexes, [Ag(phendione)_2_]ClO_4_ and [Cu(phendione)_3_](ClO_4_)_2_.4H_2_O (henceforth represented as their biologically active cations [Ag(phendione)_2_]^+^ and [Cu(phendione)_3_]^2+^), affected crucial physiological events of *P. verrucosa* (Granato et al., 2017). These three agents inhibited fungal proliferation, presenting minimum inhibitory concentration [MIC; mg/L (μM)] values of 2.5 (12.0), 2.5 (4.0), and 5.0 (5.0), respectively. Moreover, the compounds affected filamentation, sterol production, and the metallo-type peptidase activity of *P. verrucosa* (Granato et al., 2017).

Cytotoxicity assays revealed that phendione, [Ag(phendione)_2_]^+^, and [Cu(phendione)_3_]^2+^ were well tolerated *in vitro* by several mammalian tumor and non-tumor lines and also macrophages, and *in vivo* by Swiss mice and *Galleria mellonella* larvae (McCann et al., 2012; Gandra et al., 2020). This insect larva is a good and alternative *in vivo* experimental model due to the similarities exhibited with respect to the innate immune system of mammals, its ease of manipulation, low cost, and ethical acceptance (Jemel et al., 2020). Thus, *G. mellonella* larvae are widely used to assess the toxicity, efficacy, and safety of conventional antifungal agents and potential new drugs (Kavanagh and Sheehan, 2018; Maurer et al., 2019; Treviño-Rangel et al., 2019; Jemel et al., 2020).

The antimicrobial efficacy of phendione and its metal-based complexes has been shown against planktonic cells and also biofilm-growing cells (Viganor et al., 2016; Gandra et al., 2017; Ventura et al., 2020). Several studies have highlighted the biofilm relevance in the fungal pathogenesis, contextualizing the real issue of this structure formation in the medical settings (Vila and Rozental, 2016; Santos et al., 2018; Wall et al., 2019). This complex microbial community, which is adhered to a surface, is surrounded by an extracellular matrix that gives its cells several advantages, such as increased virulence, immune system, and environmental stress protection as well as resistance to antimicrobial agents (Costa-Orlandi et al., 2017; Santos et al., 2018). The biofilm extracellular matrix is formed mainly by polysaccharides, but also contains proteins, lipids, and DNA. This matrix is one of the most relevant structures of the biofilm and is directly associated with antimicrobial resistance (Ramage et al., 2012; Santos et al., 2018). In recent years, the capability of filamentous fungi (e.g., *Aspergillus fumigatus*, *Paracoccidioides brasiliensis*, *Scedosporium* spp., and *Exophiala dermatitidis*) to form a biofilm has been well documented (Kaur and Singh, 2014; Sardi et al., 2015; Mello et al., 2016; Kirchhoff et al., 2017). The ability of *P. verrucosa* to establish a biofilm on a polystyrene surface was previously investigated by our research group, and it was found that biofilm-growing *P. verrucosa* cells were more resistant to the action of conventional antifungal drugs than their planktonic counterparts (unpublished data).

The present study was designed to investigate the effects of phendione, [Ag(phendione)_2_]^+^, and [Cu(phendione)_3_]^2+^ on *P. verrucosa*, focusing on (i) the combination of these test agents with the classical antifungal drug, amphotericin B, (ii) biofilm formation and disarticulation, (iii) the *in vitro* interaction with human macrophages; and (iv) *in vivo* infection using *G. mellonella* larvae as a model.

## Materials and Methods

### Fungal Growth Conditions

*P. verrucosa* (strain FMC.2214 isolated from a human patient with chromoblastomycosis and used in our previous work; Granato et al., 2017) was maintained in Sabouraud dextrose agar (SDA) medium with mineral oil at 4°C. For all assays, fungal cells were cultivated for 7 days under constant agitation (130 rpm) at 26°C in 100 ml of Czapek-Dox broth medium (BD-Difco, United States). Conidia were obtained by gauze filtering followed by centrifugation at 4,000 × *g* for 10 min. The fungal cells were then washed three times with saline solution (0.85% NaCl), and the number of conidia cells was estimated using a Neubauer chamber (Granato et al., 2015).

### Test Compounds

Phendione was purchased from Sigma-Aldrich. [Ag(phendione)_2_]^+^ and [Cu(phendione)_3_]^2+^ were prepared by reacting phendione with the appropriate metal perchlorate salts in accordance with procedures previously published by McCann et al. (2004). For all of the experiments, the compounds were dissolved in dimethyl sulfoxide (DMSO; Sigma-Aldrich).

### Combinatory Effects of Phendione and Its Metal Complexes With Amphotericin B on *P. verrucosa* Viability

The drug combinations were assessed using the checkerboard microdilution method as described by Chudzik et al. (2019). Briefly, initial concentrations equivalent to 8 × MIC of amphotericin B (AmB, MIC = 6.25 mg/L) were serially diluted in a cross fashion mode against 4 × MIC of each compound (phendione, [Ag(phendione)_2_]^+^, and [Cu(phendione)_3_]^2+^) in 96-well plates containing Roswell Park Memorial Institute (RPMI) 1640 medium (Sigma-Aldrich). Thus, the final concentrations range from 0.09 to 50 mg/L for AmB, 0.039 to 20 mg/L for [Cu(phendione)_3_]^2+^, and 0.019 to 10 mg/L for both phendione and [Ag(phendione)_2_]^+^. The inoculation and culture conditions followed the Clinical and Laboratory Standards Institute (CLSI, 2008) standard document M38-A2 for filamentous fungi with some minor modifications (Granato et al., 2017). Minimum inhibitory concentration values were established when the combination of AmB and each test compound inhibited 100% of the fungal growth as evidenced by visual inspection and the resazurin staining assay (Liu et al., 2007). The interaction type of two-drug combination was defined according to the fractional inhibitory concentration index (FICI) from the following formula: FICI = FICA + FICB, where FICA and FICB = MIC of drug A or B in combination divided by the MIC of drug A or B alone. Synergism was established when FICI value ≤ 0.5; additivity FICI > 0.5 to < 2.0; indifference FICI ≥ 2.0 to <4.0; and antagonism FICI ≥ 4.0 (Chudzik et al., 2019).

### Effects of Phendione and Its Metal Complexes on *P. verrucosa* Biofilms

In this set of experiments, phendione, [Ag(phendione)_2_]^+^ and [Cu(phendione)_3_]^2+^ were tested on *P. verrucosa* biofilm using the broth microdilution assay in accordance with the document M38-A2 CLSI (2008) and Mello et al. (2016). Firstly, the compounds were examined for their ability to modulate biofilm formation. Conidia (1 × 10^6^ cells) were placed in flat-bottom 96-well polystyrene microtiter plates containing RPMI medium supplemented or not with each test compound at concentrations varying from MIC to ⅛ × MIC values. After 72 h, non-adhered fungal cells were removed and the plates containing adhered biofilm-growing cells were subjected to colorimetric assays designed to measure the following biofilm parameters: biomass, viability, and extracellular matrix. The biomass quantification was evaluated after biofilm fixation with methanol (at 99%), followed by staining for 20 min with 0.3% crystal violet solution and the absorbance read at 590 nm. The determination of metabolic activity was conducted in non-fixed biofilms by the addition of 100 μl of a solution containing 2,3-bis (2-methoxy-4-nitro-5-sulfophenyl)-5-[(phenylamino) carbonyl]-2H-tetrazolium hydroxide (XTT, 0.04 mg) and menadione (0.0005 mg) into the plate wells, followed by incubation in the dark at 37°C for 4 h, and finally an absorbance reading at 490 nm. The assessment of the extracellular matrix was carried out using non-fixed biofilms, by staining with 0.1% safranin solution and the absorbance read at 530 nm (Mello et al., 2016). In order to investigate the effect of phendione and its metal complexes on mature biofilms, *P. verrucosa* conidia (1 × 10^6^ cells) were added to 96-well polystyrene microplates and incubated for 72 h. Then, different concentrations of the test compounds, ranging from MIC to 64 × MIC values, were added to the plates and these were incubated for an additional 48 h. The biofilm MIC_90_ value (bMIC_90_) was determined by considering the lowest concentration of each test compound capable of causing a 90% reduction in biofilm viability (Romera et al., 2019). System controls were prepared with non-treated cells in RPMI medium supplemented or not with DMSO, and RPMI medium containing DMSO or not. All dyes and reagents used for the colorimetric assays were obtained from Sigma-Aldrich. The absorbance measurements were performed using a microplate reader (SpectraMax M3, Molecular Devices, United States). Biofilm formation was observed using an inverted microscope (Nikon TS100-F, Tokyo, Japan) with a ×40 objective lens.

### Effects of Phendione and Its Metal Complexes on *P. verrucosa*-Macrophage Interaction

#### Cell Culture and Stimulation

Human monocytic leukemia THP-1 cells (ATCC TIB-202) were cultured in 25 cm^3^ culture flasks containing RPMI 1640 medium and 10% fetal bovine serum (Sigma-Aldrich) at 37°C in a 5% CO_2_ atmosphere. For experimental assays, 1 × 10^6^ cells were added to each well of a 24-well cell culture plate and cultivated in the same conditions as detailed above, with the addition of phorbol myristate acetate (PMA, 80 nM, Sigma-Aldrich) to promote the differentiation of monocytic cells into macrophages. After 24 h, the THP-1 cells were washed three times with RPMI and incubated in fresh medium without PMA for an additional 24 h to allow for cell recovery. The differentiation of THP-1 monocytes into macrophages was confirmed by observing the cells adhesion capacity to culture plates and alteration of their morphology to a flat, amoeboid, and spreading shape using inverted microscope Nikon (Batista-Silva et al., 2016).

#### Fungi-Host Cells Interaction: Pre-treatment Assay

Conidia (2 × 10^6^ cells) were treated with each test compound at concentrations varying from ¼ × MIC to 2 × MIC values, and after 20 h at 35°C, the fungal viability was assessed using the XTT assay (Mowat et al., 2007). For the interaction assay, *P. verrucosa* conidia were treated for 20 h with a non-cytotoxic concentration of each test compound, washed, and incubated for 30 min with the fluorescent dye PKH26 (2 μM; Sigma-Aldrich). After that, 250 μl of fetal bovine serum were added to interrupt the reaction, and the conidial cells were then washed and re-suspended in RPMI medium and incubated with macrophages in a 10:1 ratio (fungi:macrophage) for 3 h in a 5% CO_2_ atmosphere at 37°C. The interaction systems were washed three times with sterile phosphate-buffered saline, pH 7.2 (PBS) and then with 2 mM PBS-EDTA buffer for their transference to cytometry tubes (Ersland et al., 2010). The interaction was monitored by flow cytometry (BD FACSAria) using Flowjo software. Results were represented as percentage of infected macrophages, which indicates the percentage of macrophage infected with fluorescent-labeled conidial cells. Systems consisting of non-treated conidia (control) and unlabeled conidia were also examined. The fluorescence values were adjusted after subtracting the non-specific fluorescence values of unlabeled cells (autofluorescence). In parallel, macrophages infected with either untreated or treated conidial cells were lysed with sterile cold water, and the suspensions were plated onto SDA medium to count the number of colony forming units (CFU; Palmeira et al., 2008).

#### Fungi-Host Cells Interaction: Post-treatment (Killing) Assay

In this set of experiments, the effect of the test compounds on macrophage viability was initially investigated. THP-1 macrophages (1 × 10^6^) were incubated in a 96-well cell culture plate for 20 h in the absence (control) or in the presence of each test compound at a concentration varying from 0.03 to 5 mg/L. Then, the viability of macrophages was assessed after addition of 3-(4,5-dimethylthiazol-2-yl)-2,5-diphenyl tetrazolium bromide (MTT, Sigma-Aldrich) at 0.5 mg/ml and incubation for 3 h at 37°C as described by Mosmann (1983). Subsequently, in order to investigate the killing capability of THP-1 macrophages against *P. verrucosa*, viable conidia were washed in PBS and transferred to a 24-well plate containing macrophages at 10:1 ratio (fungi:macrophage) and incubated for 1 h at 37°C. Non-adhered conidia were removed and the interaction systems were incubated for 20 h with RPMI medium supplemented or not (control) with a non-cytotoxic concentration of test compound. The cultures were washed with PBS, lysed with sterile cold water and the suspensions then plated onto SDA medium for establishing the CFU number (Palmeira et al., 2008).

### Effect of [Ag(phendione)_2_]^+^ on *G. mellonella* Larvae Infected With *P. verrucosa*

#### *G. mellonella* Larvae

The larvae of *G. mellonella* were reared in the insectary of the Department of Biochemistry of the Institute of Chemistry at UFRJ in plastic boxes at 21°C in the dark. Healthy larvae from the last instar with similar size (15–20 mm) and weight (0.2 g), and without alterations in their color, were chosen for the experiments. In order to determine the *in vivo* cytotoxicity and the antifungal activity of each test compound, 10 larvae were used in each experimental system (McCann et al., 2012; Fernandes et al., 2017).

#### Larval Survival Assay

Firstly, the non-toxic concentrations of [Ag(phendione)_2_]^+^ and the appropriate fungal cellular density were determined as described by McCann et al. (2012). Briefly, the compound (10 μl) was administered by injection through the last left proleg directly into the larvae haemocoel, using a Hamilton syringe, resulting in the final concentrations of either 5 or 10 μg compound/larva. Simultaneously, *P. verrucosa* conidia were suspended in PBS and adjusted to final densities equal to 1 × 10^4^, 1 × 10^5^, 1 × 10^6^, 4 × 10^6^, or 1 × 10^7^ and inoculated as described above into each group of *G. mellonella* larvae. Then, the larvae were incubated at 37°C for 7 days and survival monitored every 24 h. Larvae mortality was assessed by lack of movement in response to mechanical stimulus. Larvae inoculated with 10 μl of PBS or DMSO were used as control systems. Infected larvae images were obtained after 1 h of incubation. For antifungal assessment, each *G. mellonella* larvae group was infected with *P. verrucosa* conidia, at a concentration presenting an intermediate rate of larvae mortality, and after 2 h a non-cytotoxic concentration of the test compound was injected. After that, the experimental protocol and the survival rate determination were performed as detailed above.

### Statistical Analysis

The experiments were performed in triplicate in three independent experimental sets. The graphics and data were constructed and analyzed statistically by means of the ANOVA-test. Survival curves of *G. mellonella* larvae experiments were constructed using the method of Kaplan-Meier and compared using the Log-Rank (Mantel-Cox) test. All statistical analyses were performed with GraphPad Prism 5.0 software. Values of *p =* 0.05 or less were assumed as significant.

## Results and Discussion

### Combinatory Effects of Phendione and Its Metal Complexes With Amphotericin B on *P. verrucosa* Viability

Using the checkerboard assay, we firstly investigated the effects of AmB combined with each of the test compounds (Table 1). The combinations, AmB-[Cu(phendione)_3_]^2+^ and AmB-[Ag(phendione)_2_]^+^, were both additive (FICI = 0.7 and 1.0, respectively). Although none of the drug combinations displayed synergism (FICI ≤ 0.5) in the present case, such an effect has been observed in previous studies involving combinations of the metal-phendione complexes with classical antifungal drugs. Eshwika et al. (2004) showed that *C. albicans* treated with non-cytotoxic concentrations of [Ag(phendione)_2_]^+^ became more susceptible to miconazole and AmB. In a later study on *C. albicans*, the combination of AmB with copper(II) ions (AmB-Cu(II) complex) was again found to be more effective than AmB alone (Gagoś et al., 2011; Chudzik et al., 2013). It has been suggested that drug combinations with an additive effect may enhance pharmaceutical efficacy and reduce toxicity when each compound targets distinct stages of the same biological pathway (Spitzer et al., 2017). Importantly, the combination of benzothioureas with caspofungin has been shown to function in an additive manner regarding anti-cryptococcal activity, even though caspofungin on its own is ineffective against *Cryptococcus* species (Hartland et al., 2016; Spitzer et al., 2017). Overall, metal-based drugs have different modes of action conventional organic antifungal agents, thus combination therapy could potentiate the effect as well as possibly minimizing both resistance and toxicity.

**Table 1:** Effects of phendione and its metal complexes combined with amphotericin B on *Phialophora verrucosa* growth.

MIC mg/L (μM)				
Agents	Alone	Combined	FIC	FICI
AmB	6.2 (6.8)	3.1 (3.4)	0.5	1.0
Phendione	2.5 (12.0)	1.2 (6.0)	0.5	
AmB	6.2 (6.8)	3.1 (3.4)	0.5	1.0
[Ag(phendione)_2_]^+^	2.5 (4.0)	1.2 (2.0)	0.5	
AmB	6.2 (6.8)	1.5 (3.4)	0.2	0.7
[Cu(phendione)_3_]^2+^	5.0 (5.0)	2.5 (2.5)	0.5	

The type of interaction was determined by calculating the fractional inhibitory concentration (FIC) index (FICI) as described by Chudzik et al. (2019) and detailed in Material and Methods. Minimum inhibitory concentration (MIC) was defined as the lowest concentration capable of inhibiting 100% of fungal growth. AmB, amphotericin B.

### Effects of Phendione and Its Metal Complexes on *P. verrucosa* Biofilms

**Figure 1:**
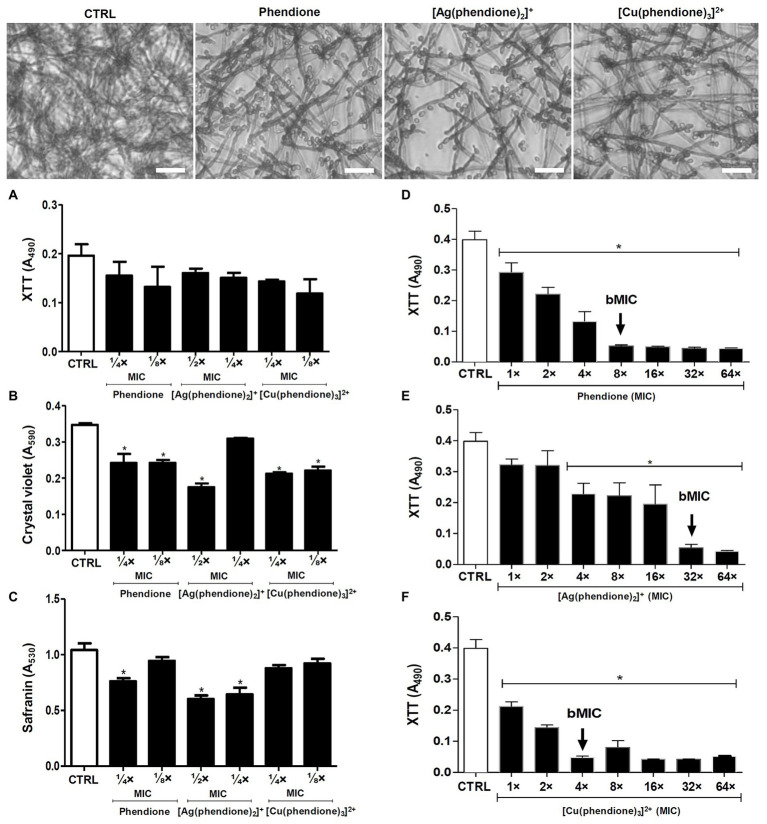
Effects of phendione and its metal complexes on *P. verrucosa* biofilm formation and maturation. Conidia (1 × 10^6^) were added to 96-well plates containing Roswell Park Memorial Institute (RPMI) medium supplemented with sub-MIC concentrations of each test compound. After incubation for 72 h at 37°C, **(A)** cell viability, **(B)** quantification of biomass, and **(C)** extracellular matrix were evaluated, as described in Material and Methods. The same conidia density **(D–F)** was placed to interact with polystyrene for 72 h in RPMI medium. Then, concentrations varying from MIC to 64 × MIC of each test compound were added and the plates incubated for additional 48 h. The MIC_90_ values of the biofilm (bMIC_90_) were detected using the XTT reduction assay. Systems containing non-treated conidia were also prepared (CTRL, control). The eluent (dimethyl sulfoxide, DMSO) did not affect fungal growth (data not shown). The symbol (^*^) highlights the MIC values that caused a statistically significant reduction on each evaluated parameter in relation to the respective control (*p* < 0.05). Representative images of biomass (crystal violet-stained) formed by *P. verrucosa* non-treated (CTRL, control) and treated with phendione (¼ × MIC) and its silver(I) (½ × MIC) and copper(II) (¼ × MIC) complexes. Bar: 20 µm.

The effectiveness of the phendione-based drugs to inhibit *P. verrucosa* conidial growth was previously described by our research group, with the following order: [Cu(phendione)_3_]^2+^ > [Ag(phendione)_2_]^+^ > phendione (Granato et al., 2017). Herein, we evaluated the effects of the test compounds upon biofilm formation by *P. verrucosa* on a polystyrene substrate. The fungal cells (1 × 10^6^) incubated with sub-MIC concentrations preserved their metabolic viability along the process of biofilm formation, as shown by the XTT assay (Figure 1A). However, the biofilm biomass was affected by the incubation with the test compounds, showing a significant reduction when treated with phendione and [Cu(phendione)_3_]^2+^ at both ¼ × and ⅛ × MIC values, as well as with [Ag(phendione)_2_]^+^ at ½ × MIC value (Figure 1B). The extracellular matrix production was also affected by [Ag(phendione)_2_]^+^ at both ½ × and ¼ × MIC values, and by phendione at ¼ × MIC (Figure 1C).

The test compounds were also evaluated regarding their ability to disturb a mature biofilm formed by *P. verrucosa*. Phendione, [Ag(phendione)_2_]^+^, and [Cu(phendione)_3_]^2+^ inhibited fungal viability by approximately 90% (bMIC_90_) when treated with concentrations [mg/L (μM)] of 20 (96), 80 (128), and 20 (20), respectively (Figures 1D–F). Moreover, biofilm cells were more resistant to the test compounds, since their bMIC_90_ values were higher than those observed for the planktonic counterparts (Granato et al., 2017), increasing by about 4-, 8-, and 32-fold to [Cu(phendione)_3_]^2+^, phendione, and [Ag(phendione)_2_]^+^, respectively. Previous studies had shown that the biofilm of the *C. haemulonii* species complex was also disturbed by phen- and phendione-containing compounds, including copper(II) and silver(I) complexes (Gandra et al., 2017). In contrast to *P. verrucosa*, silver(I) chelates (16 mg/L, 25.5 μM) had a lower geometric mean of the bMIC values than their copper(II) counterparts (27.4 mg/L, 28.4 μM) for the *C. haemulonii* species complex. However, in comparison to the metal complexes containing perchlorate counter anions, the most active were manganese(II), silver(I), and copper(II) phen chelates containing coordinated dicarboxylate anions, which presented bMIC_50_ values below 10 μM for these opportunistic yeasts (Gandra et al., 2017).

### Effects of Phendione and Its Metal Complexes on the Interaction Between *P. verrucosa* and Human Macrophages

**Figure 2:**
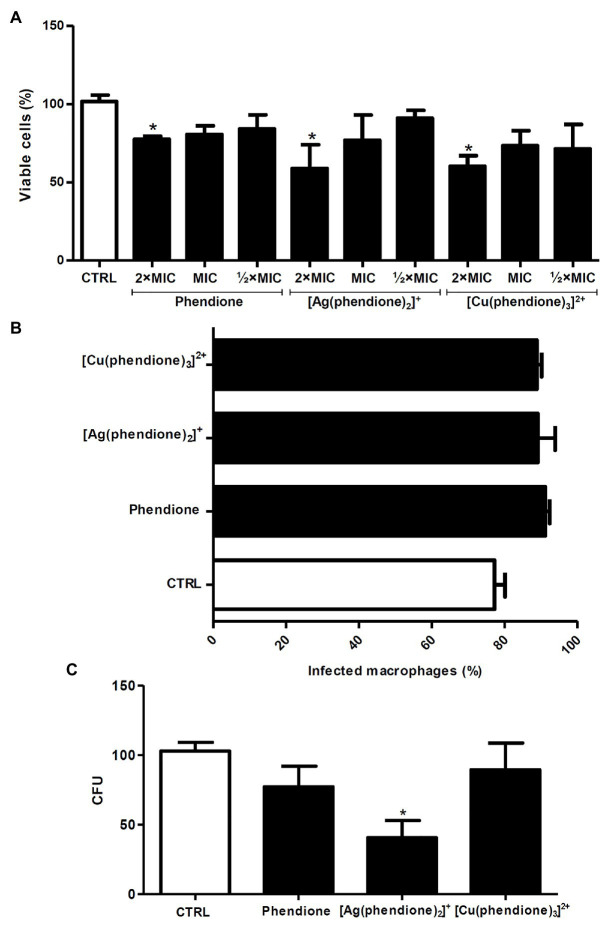
Effects of phendione and its metal complexes on *P. verrucosa*-macrophage interaction. **(A)** Conidia (2 × 10^6^) were treated for 20 h with each test compound in order to establish non-cytotoxic concentrations using the XTT assay. Then, the fungal cells **(B,C)** were treated with the MIC values of each compound, washed, and added to 24-well plates containing macrophages, in a 1:10 ratio (macrophage:fungi). After 3 h at 37°C, **(B)** the cells were transferred to tubes and subjected to flow cytometry to determine the number of infected macrophages, **(C)** the cells were washed, lysed with sterile cold water and the colony forming units (CFU) counted. Systems containing non-treated conidia were also prepared (CTRL, control). The eluent (DMSO) did not affect fungal growth (data not shown). For the flow cytometry assay, conidia were stained with PKH26. The values represent the mean SD of three independent experiments performed in triplicate. Asterisks indicate values of *p* ≤ 0.05.

Firstly, *P. verrucosa* conidial (2 × 10^6^) viability was assessed after treatment with different concentrations of the test compounds. The XTT assay showed that only the highest concentration (2 × MIC) affected fungal viability after 20 h (Figure 2A). The pre-treatment of *P. verrucosa* conidia with the MIC value of each compound did not significantly affect the percentage of infected macrophages (Figure 2B). However, the number of intracellular viable conidia was significantly diminished (around 60%) when the fungal cells were pre-treated with [Ag(phendione)_2_]^+^, as judged by the CFU assay (Figure 2C).

**Figure 3:**
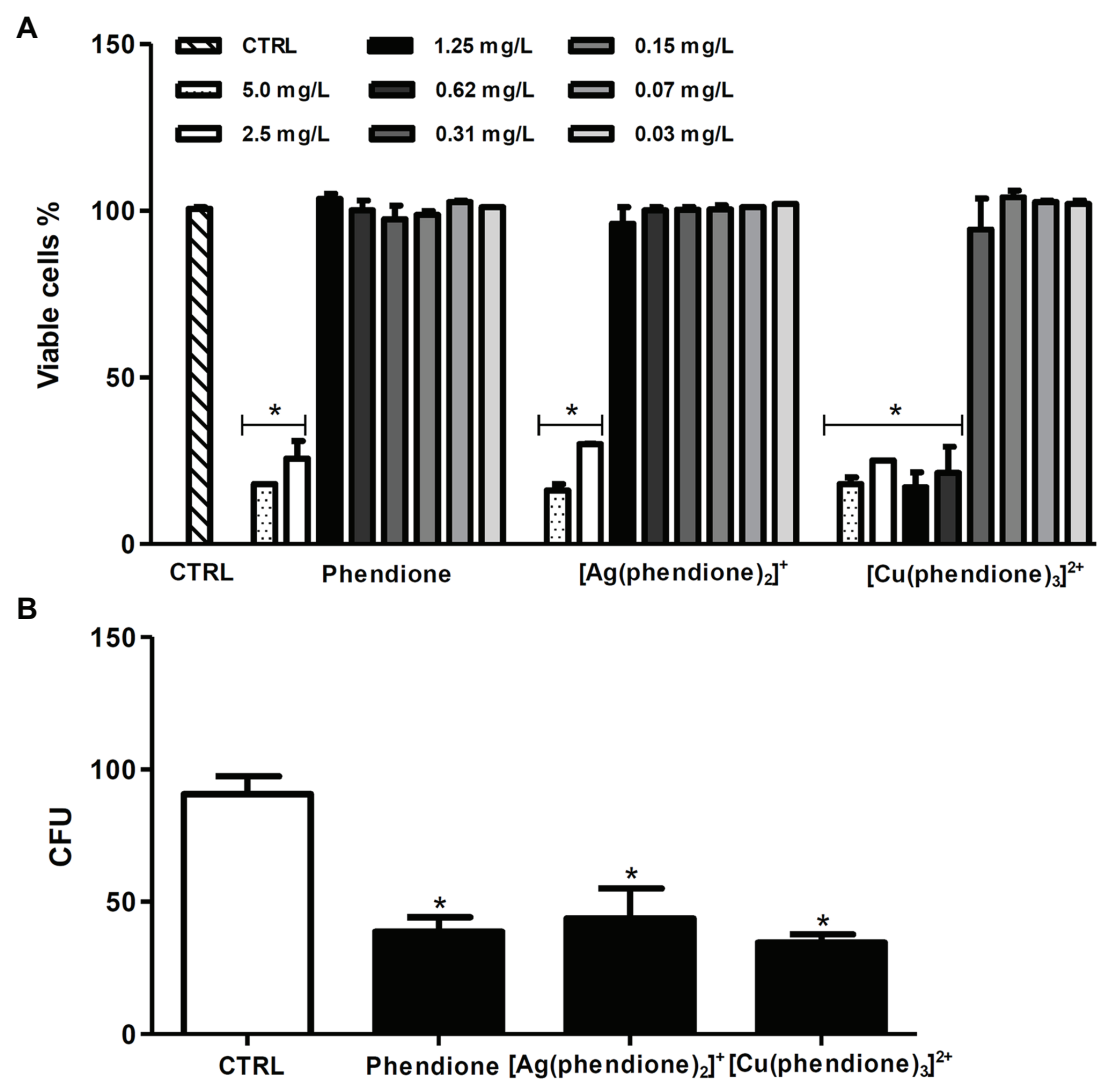
Effects of phendione and its metal complexes on THP-1 viability and the ability of macrophages to kill *P. verrucosa* conidia. **(A)** Macrophages (1 × 10^6^) were incubated in the absence (CTRL, control) or in the presence of phendione and its silver(I) and copper(II) complexes (0.03–5 mg/L) for 20 h. After treatment, macrophage viability was determined using the MTT assay. **(B)** THP-1 cells were infected with *P. verrucosa* conidia in a ratio of 1:10 (macrophage:fungi) for 1 h. Then, non-adherent fungi were removed and the microplates incubated for an additional 20 h with non-cytotoxic concentrations [mg/L (μM)] of phendione [1.25 (6)], [Ag(phendione)_2_]^+^ [1.25 (2)], and [Cu(phendione)_3_]^2+^ [0.31 (0.31)]. Then, macrophages were washed and lysed with sterile cold water, and the suspensions were plated onto sabouraud dextrose agar (SDA) medium to count the number of colony forming units (CFU). The values represent the mean SD of three independent experiments performed in triplicate. Asterisks indicate values of *p* ≤ 0.05.

In order to investigate the killing capability of macrophages against *P. verrucosa* during the *in vitro* treatment, we firstly evaluated the effect of the test compounds on macrophage viability using the MTT assay. The results showed that phendione and its silver(I) complex were less toxic than the copper(II) complex, since around 90% of the macrophages remained viable following treatment with 1.25 (6 μM), 1.25 (2 μM), and 0.31 mg/L (0.31 μM) concentrations, respectively (Figure 3A). Similarly, previous cytotoxicity studies showed that animal cell lineages were more tolerant toward [Ag(phendione)_2_]^+^. McCann et al. (2012) showed that [Ag(phendione)_2_]^+^ (IC_50_ > 10 mg/L; 15.9 μM) was less toxic than [Cu(phendione)_3_]^2+^ (IC_50_ = 6.1 mg/L; 6.3 μM) when murine macrophages (RAW 264.7 lineage) were assayed. In addition, Gandra et al. (2017) assessed the toxicity of the same compounds against A549, a lung epithelial cell lineage, and showed that the CC_50_ value was significantly larger for the silver(I) complex (3.76 mg/L) than its copper(II) analogue (0.68 mg/L). Herein, we showed that the infected macrophages, subsequently treated for 20 h with non-toxic concentrations of phendione [1.25 mg/L (6 μM)], [Ag(phendione)_2_]^+^ [1.25 mg/L (2 μM)], and [Cu(phendione)_3_]^2+^ [0.31 mg/L (0.31 μM)], were able to reduce the viability of intracellular conidial cells by approximately 65, 60, and 70%, respectively (Figure 3B).

The mechanism of action of metal complexes of phen and phendione has not yet been fully established. However, the impact of these compounds on fungal cells can be attributed to their ability to induce different cellular changes, such as disruption of the cell membrane, rupture of internal organelles, nuclear fragmentation, sequestration of essential metals, and damage of the mitochondrial function (Coyle et al., 2003; McCann et al., 2012). In the previous study involving *P. verrucosa* conidia, we revealed some potential mechanisms of action of these test compounds (Granato et al., 2017). The phendione metal complexes were able to affect the production of fungal membrane ergosterol, thus possibly contributing to cell death. In addition, the complexes also inhibited the metallopeptidase activity of *P. verrucosa* conidia (Granato et al., 2017). Inhibition of this enzyme can prevent the fungal cells ability to access peptides and amino acids essential for their nutrition and thus restricting cell growth. From the current study, it is evident that addition of the test compounds improved the antifungal action of macrophages. The increase of compound-treated *P. verrucosa* susceptibility to macrophage killing could be related to an increase in the production of reactive oxygen species by these phagocytes since the respiratory burst is one of the most important mechanisms for the antimicrobial immunity of phagocytic cells (Herb and Schramm, 2021). Thus, the enhanced ability of macrophages to combat the fungal infection could be explained by the direct action of the test compounds on the intracellular conidia, and by the induction of intracellular macrophage mechanisms that mediate the antimicrobial immune responses, just as has previously been reported in regard to the anti-mycobacterial and antitumoral activities of phen- and phendione-based complexes (Kellett et al., 2011; McCarron et al., 2018). However, further studies are required in order to more fully understand the mechanisms involved in the anti- *P. verrucosa* activity of the test compounds.

### Effects of [Ag(Phendione)_2_]^+^ on *G. mellonella* Infected With *P. verrucosa*

**Figure 4:**
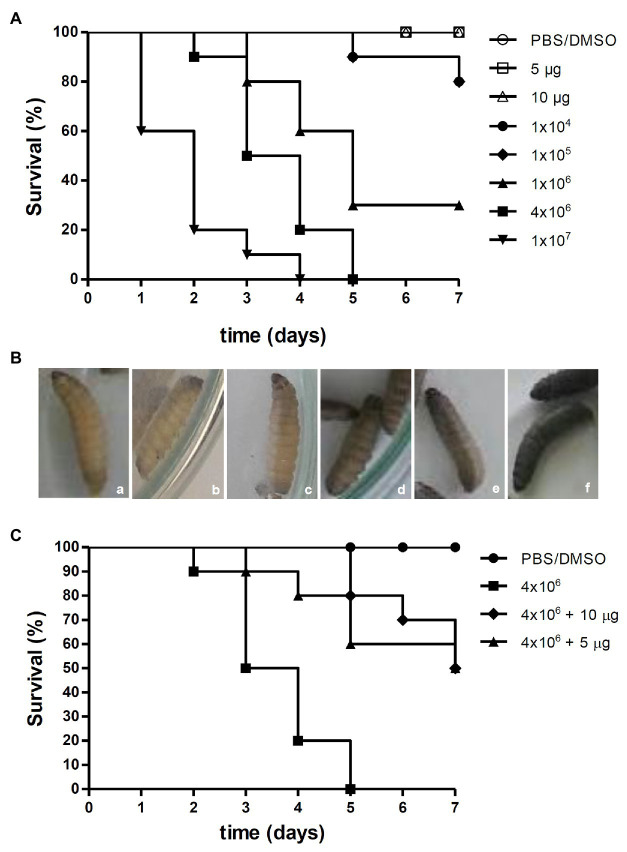
Effects of [Ag(phendione)_2_]^+^ on *P. verrucosa* infection of *G. mellonella* larvae. **(A)** Different cell densities of *P. verrucosa* conidia and [Ag(phendione)_2_]^+^, at concentrations of 5 and 10 μg/larva, were injected in *G. mellonella* larvae and the survival rate determined over a total period of 7 days. **(B)** Representative images of *G. mellonella* larvae 1 h post injection with (a) PBS/DMSO and different densities of *P. verrucosa* conidia (b) 1 × 10^4^, (c) 1 × 10^5^, (d) 1 × 10^6^, (e) 4 × 10^6^, and (f) 1 × 10^7^. **(C)** Each larva was infected with *P. verrucosa* conidia (4 × 10^6^), and after a period of 2 h was treated with the silver(I) complex at a concentration of either 5 or 10 μg/larva. Injection with only the PBS/DMSO eluent represents the control. The survival rate (10 larvae/systems) was monitored over 7 days, and death was assessed by the lack of larval movement in response to a stimulus.

Although the copper(II) chelate was the most effective in disturbing a *P. verrucosa* mature biofilm, the silver(I) complex was less toxic toward THP-1 and it also affected the viability of conidial cells, at both pre- and post-treated proposals, in the *in vitro* interactions with macrophages. Thus, [Ag(phendione)_2_]^+^ was selected for the *in vivo G. mellonella* experiments. At dosages of 5 and 10 μg per larva, the silver(I) complex did not affect *G. mellonella* viability for a test period of up to 7 days (Figure 4A). This result corroborated the data obtained previously by McCann et al. (2012), who showed the low toxicity of phendione and its metal complexes for an exposure period of 72 h using the *G. mellonella* larvae as an experimental model. Similarly, the test compounds were also well tolerated by Swiss mice, as previously reported by McCann et al. (2012). In that study, phendione, [Ag(phendione)_2_]^+^, and [Cu(phendione)_3_]^2+^, at dosages up to 45 mg/kg, were injected into mice and after 7 days no deaths were recorded. Interestingly, only [Ag(phendione)_2_]^+^, administered at a concentration of 150 mg/kg over the same time period, did not cause mortality, indicating that this complex was the least toxic. Thus, mice tolerance was in the order [Ag(phendione)_2_]^+^ > phendione > [Cu(phendione)_3_]^2+^ (McCann et al., 2012).

The survival of *G. mellonella* larvae following infection with *P. verrucosa* was also studied (Figure 4A). After inoculation of larvae with the lowest fungal densities (1 × 10^4^ and 1 × 10^5^), 80% survived up to the last day of monitoring. However, 1 × 10^6^ conidia induced larvae death gradually until the fifth day, and at the end of the incubation period only 30% survived. Administering a cell density of 4 × 10^6^ conidia caused a reduction of 50% of viable larvae by the third day and 100% mortality by the fifth day. In addition, it was found that injection of 1 × 10^7^ conidia triggered 80% of larval death by the second day and 100% by the fourth day (Figure 4A). These experiments demonstrated that the mortality of *G. mellonella* infected with *P. verrucosa* was dependent upon the fungal inoculum density. It is well known that disease outcomes are influenced by the quantity of the infective particles. However, tests using the same invertebrate model infected with *Paracoccidioides lutzii* and *Histoplasma capsulatum* revealed that these fungi were lethal to the larvae and without any correlation with cellular density (Thomaz et al., 2013). With the current infection assay using *P. verrucosa*, it was observed that the melanization of the larvae increased in accordance with dispensed cell density. In fact, larvae inoculated with the highest *P. verrucosa* cell density (1 × 10^7^) immediately became melanized, whereas with cell densities of 1 × 10^6^ and 4 × 10^6^, the pigmentation was observed only after 1 h (Figure 4B). Larvae melanization, when exposed to other fungi, has been well documented (Li et al., 2013; Thomaz et al., 2013; Gandra et al., 2020). Like *P. verrucosa*, other non-*albicans Candida* species induced early larvae melanization and its progression was dependent upon inoculum density (Silva et al., 2018). However, premature larvae melanization was not observed following infection with *Cryptococcus neoformans* (Trevijano-Contador et al., 2015). The *G. mellonella* model was also used for studying the virulence of other fungi that cause chromoblastomycosis and phaeohyphomycosis, such as *Fonsecaea* spp. and *Exophiala jeanselmei* (Vicente et al., 2017; Huang et al., 2018). Huang et al. (2018) showed that the increase in fungal inoculum resulted in greater larval mortality. In contrast to *P. verrucosa*, the authors observed that *F. monophora* (1 × 10^6^ conidia/larva) infection induced *G. mellonella* melanization only on the second day post injection. *G. mellonella* larvae melanization process occurs in response to trauma or microbial cell invasion through the activation of phenoloxidase (Trevijano-Contador and Zaragoza, 2019). The insect enzymatic system can be activated during the immune response after recognition of specific components, such as β-glucans in the fungal cell wall (Pereira et al., 2018; Trevijano-Contador and Zaragoza, 2019).

Addition of [Ag(phendione)_2_]^+^ at two non-toxic concentrations (5 and 10 μg/larva) to *G. mellonella* larvae which were pre-infected with *P. verrucosa* (inoculum density of 4 × 10^6^ conidia), resulted in an intermediate rate of larval mortality. The results showed that by the third day of incubation, 50% of untreated larvae had perished, while 90% of larvae treated with the same two concentrations of the silver(I) complex remained alive (Figure 4C). On the fifth day, 100% mortality was recorded for the untreated larvae. However, 80% of larvae survived after [Ag(phendione)_2_]^+^ treatment at 10 μg per larva. At the lower concentration of added [Ag(phendione)_2_]^+^ (5 μg per larva) there was a 60% survival rate, suggesting a dose-dependent effect. It was also found that, following treatment with [Ag(phendione)_2_]^+^, 50% of larvae survived until the last day of monitoring (Figure 4C), thus clearly demonstrating a protective effect by the silver(I) complex. In their study of *G. mellonella* infected with *Candida* spp., Rowan et al. (2009) found that the larvae survival rate was increased after treatment with the silver(I) complex, [Ag_2_(mal)(phen)_3_] (mal H_2_ = malonic acid). Gandra et al. (2020) showed that different phen and phendione metal complexes, including [Ag(phendione)_2_]^+^, were able to inhibit *C. haemulonii* proliferation in *G. mellonella* infected larvae. In that study, the manganese(II) phen chelate containing the 3,6,9-trioxaundecanedioate dianion was the most effective. The antifungal efficacy against other fungi, such as *Candida* spp., *Cryptococcus* spp., *Aspergillus* spp., and *Trichosporon* spp. has also been assessed using the *G. mellonella* model (Kavanagh and Sheehan, 2018; Jemel et al., 2020). Thus, our present work has demonstrated that the *G. mellonella in vivo* model can be employed successfully to evaluate the antifungal effectiveness of phendione complexes presenting *in vitro* anti-*P. verrucosa* activity.

## Conclusion

The silver(I) phendione complex, [Ag(phendione)_2_]^+^, is capable of reducing the viability of *P. verrucosa* conidia following interaction with human macrophages *in vitro*, and also conferring protection, *in vivo*, to *G. mellonella* larvae infected with the fungus. Transition metal complexes represent a novel group of antifungal agents due to their multi-modal activity, low cost, ease of synthesis, and their relatively high tolerance in *in vitro* animal cells as well as in lower mammals and insects used routinely as *in vivo* experimental models. Further studies are ongoing in an effort to understand the mechanisms of action of these metal complexes against *P. verrucosa* and other fungi that also cause chromoblastomycosis and phaeohyphomycosis. This advancement will further inform and facilitate the development of more effective compounds with reduced toxicity and enhanced potency against the microbes responsible for these debilitating diseases.

## Data Availability Statement

All datasets generated for this study are included in the article/supplementary material.

## Author Contributions

MG, MDP, MCP, MM, MD, MB, AS, and LK conceived and designed the study and wrote and revised the paper. MG, TM, RN, and TR performed the experiments. All authors analyzed the data. MDP, MCP, MM, MD, MB, AS, and LK contributed the reagents, materials, and/or analysis tools. All authors contributed to the research, approved the final version of the manuscript, and agree to be accountable for all aspects of the work.

### Conflict of Interest

The authors declare that the research was conducted in the absence of any commercial or financial relationships that could be construed as a potential conflict of interest.
